# Psycho-Linguistic Changes Associated With Historical Celebrities in Henan Using Classical Chinese Big Data

**DOI:** 10.3389/fpsyg.2021.648677

**Published:** 2021-09-14

**Authors:** Yanan Zhao, Fugui Xing, Miaorong Fan, Hua Li, Tingshao Zhu

**Affiliations:** ^1^Institute of Psychology, Chinese Academy of Sciences, Beijing, China; ^2^Department of Psychology, University of Chinese Academy of Sciences, Beijing, China; ^3^Institute of QiLu-Culture Studies, Shandong Normal University, Jinan, China

**Keywords:** psycho-linguistic changes, Henan historical celebrities, CC-LIWC, classical Chinese big data, classical Chinese words segmentation system

## Abstract

Chinese civilization has a long history, and the Central Plains, with Henan as the core, is one of its birthplaces. Understanding the psycho-linguistic changes in Henan is of great significance for understanding the evolution and formation of national cultural psychology. The traditional method is mainly qualitative or speculative, which makes the consistency among the research results low. Moreover, because most of the research are conducted in a specific period or on a specific figure, they are relatively scattered and unsystematic. To systematically and quantitatively study the psychological changes in the Central Plains represented by Henan Province, this article aimed to examine the self-reported discourses of historical celebrities in Henan in official history and their psycho-linguistic changes based on the classical Chinese Linguistic Inquiry and Word Count (classical Chinese LIWC, CC-LIWC) psycholinguistic dictionary and the classical Chinese words segmentation system, using big data. The research found that the frequency of male words in Henan historical celebrities (*F* = 2.938, *p* < 0.05), of differ words (*F* = 4.767, *p* < 0.01), of motion words (*F* = 4.042, *p* < 0.01), and of time words (*F* = 5.412, *p* < 0.01) are significantly different among the five dynasties. The conclusion is that during the Spring and Autumn and the Warring States periods, 100 schools of thought contended, and the status of “scholars” in Henan rose. The frequency of male and differ words was at that point significantly higher than during the other dynasties. From “The Contention of a Hundred Schools of Thought” to “The Supremacy of Confucianism,” the scholars in Henan were in decline, and differential cognitive tendencies had diminished since the Han dynasty. During the period of the Three Kingdoms and Jin and Southern and Northern Dynasties, political powers and territories changed, and the historical celebrities in Henan show a remarkable tendency that were related to time and space. The psycho-linguistic changes found in this study are highly consistent with the development of social history, which indicates that the guidance of the social, political, and cultural environment has an important influence on the psycho-linguistic changes of social class. This is the first time that the text analysis system has been applied in classical Chinese to carry out quantitative research on the psycho-linguistic changes of ancient Chinese people, which provides new ideas and new methods for humanistic research.

## Introduction

The Central Plains is the region with the longest history in China, and it is the historical cradle of Chinese civilization. In the pluralistic and integrated pattern of Chinese civilization, the Central Plains culture plays an irreplaceable central role compared with the culture of other regions and undoubtedly plays a leading role (Fan, 2006). The academic circle has reached a consensus on the narrow sense of the “Central Plains.” The area is generally regarded as Henan Province today, excluding other regions (Han, 2012a). The historical celebrities have been the creators and inheritors of Chinese culture for thousands of years and have played an important role in inheriting and spreading the culture of China during the long history of development (Sui, 2019). The psycho-linguistic changes of Henan historical celebrities were inevitably affected by the historical social changes and social ideologies that had a certain uniqueness and significance. Therefore, studying how the psycho-linguistic characteristics of historical celebrities in Henan changed is of great significance for understanding Chinese psychological development.

In the previous psychological studies of historical celebrities, more traditional methods have been used, such as questionnaire surveys, personality assessments, and case analyses. For example, researchers in the field of modern and contemporary literature have studied the personality characteristics of Xun Lu using a large collection of LOTS data, the “Eysenck Personality Questionnaire (EPQ),” and the “Meyers Briggs Personality Type Questionnaire (MBTI)” literature (Zhu, 2006). Furthermore, in the field of historical psychology, the personality of Yiqi Mei has been studied using the personality adjective rating method (Wu and Xue, 2008). Finally, the researchers have used psychobiographical methods to analyze the prime ministers through the ages and have identified four of the personality traits of Liang Zhuge as loyalty, wisdom, resolve, and prudence (Shu, 2014). The limitation of the above research is the small research sample sizes, which are not conducive to a long-term longitudinal comparative study, and the high costs of questionnaire surveys, manpower, and material resources. In addition, the previous studies have emphasized qualitative analysis more than quantitative analysis.

Words and languages are the core of psychology and communication. They are the medium by which cognitive, personality, clinical, and social psychologists attempt to understand human beings (Tausczik and Pennebaker, 2009). Vocabulary provides information about social processes, e.g., who has higher social status, whether a team cooperates well, whether someone is lying, and the quality of intimate relationships among the people. The choice of words provides information about human perception (Semin and Fiedler, 1988). The prepositions (such as to, with, and above), cognitive mechanisms (such as cause, know, and think), and words are longer than six letters that all indicate more complex local area network languages (LAN) (Hartley et al., 2003). The higher the frequency of emotional vocabulary use is, the stronger the immersion in the traumatic event is, which strengthens the experience of physical pain (Holmes et al., 2007). Since the early 1990s, through the development of the computer program LIWC—language query and word count calculation (Pennebaker et al., 2007),—we have witnessed a new generation of text analysis from computer science and computational linguistics. The above research shows that the LIWC computer program provides a new research method for the study of psycho-linguistic changes.

In nearly 5,000 years of Chinese history, many ancient documents have accumulated, which have preserved and precipitated Chinese spiritual civilization and Chinese history and culture. These recoded thoughts, words, and behaviors of ancient people provide the possibility to study the historical celebrities. Although ancient Chinese also belongs to the Chinese, it is significantly different from modern Chinese in morphology and syntax. Moreover, modern Chinese has strong logic, while ancient Chinese has a quite flexible syntax. In terms of genre, there are prose, rhyme, parallel prose, etc. In terms of syllables, ancient Chinese vocabulary is classified into monosyllabic words, two-tone words, and compound words. Before the May Fourth Movement (May 4, 1919), all ancient Chinese classics were written without punctuation; thus, “clear sentence reading” with punctuated sentences is the precondition for people to understand ancient Chinese. In ancient times, sentence reading was quite complicated. Reading the text first, it was necessary to use function words, analyze the sentence patterns, use grammar, and refer to the ancient annotations to divide an article into sentences. Ancient sentence reading was a process of artificial clauses. In designing the word segmentation algorithm of ancient Chinese, we must consider the characteristics of ancient Chinese vocabulary and the data structure of the relevant corpus dictionary. In ancient Chinese, monolingual words account for the majority, and their grammar, morphology, and syntactic structure are complex and there are often certain changes in ancient texts at different times. Therefore, ancient Chinese word segmentation is a huge undertaking that integrates ancient Chinese grammar, history, mathematics, and computer science (Yang, 2007). There is no independent distinguishing mark for words in Chinese that needs to be judged by the model itself. Ancient Chinese word segmentation mainly faces the following four difficulties: (1) Due to the flexible and diverse use of ancient Chinese, many ancient Chinese words have multiple meanings that often lead to ambiguity. (2) The concept of ancient Chinese words is not clear. In many places, the division is ambiguous, and there is no normative standard. (3) In the use of ancient Chinese, there are few commonly used words, but these words eventually constitute a huge vocabulary, and with the promotion of the Internet, there is still a steady stream of new words. (4) There are too many ancient Chinese words, resulting in the appearance of many unregistered words. The model often encounters unregistered words in word segmentation; therefore, it is difficult to deal with them (Wang, 2018). Although ancient Chinese participles are difficult, ancient Chinese texts are a rich source of information. Most of the cultural history of the Chinese nation in China is contained in various works from the past dynasties, and these works are almost all written in ancient Chinese. If we want to have a deep understanding of the history, culture, and characters of the Chinese nation and draw information from ancient literature, we must study ancient Chinese. Therefore, the students in the research group used a large-scale ancient Chinese corpus to build an ancient Chinese word segmentation dictionary through big data technology and applied positive maximum matching to achieve word segmentation of classical Chinese. Compared with the two-word segmentation modes of Jia Yan, this model has obvious improvements in indicators, such as accuracy rate, recall rate, and F value (frequency) and has achieved good results in general.

The Chinese civilization has a long history, and the Central Plains, with Henan as the core, is one of its birthplaces. Understanding the psycho-linguistic changes in Henan is of great significance for understanding the evolution and formation of national cultural psychology. The traditional method is mainly qualitative or speculative, which makes the consistency between the research results low. Moreover, because most of the research is conducted in a specific period or on a specific figure, it is relatively scattered and not systematic enough. To conduct a systematic and quantitative study on the psycho-linguistic changes in the Central Plains represented by Henan using classical Chinese big data, this article took the self-reported discourses of historical celebrities in Henan in official history and researched their psycho-linguistic changes based on the classical Chinese LIWC psycho-linguistic dictionary (classical Chinese LIWC, CC-LIWC) (Fan, 2020) and the classical Chinese words segmentation system (Xing and Zhu, 2021).

Study the laws and phenomena of psycho-linguistic changes along with social changes in the process of historical development and the guidance and influence of society, to provide ideas and methods for subsequent social management, social guidance, and social stability. The historical celebrities passed away long ago. Thus, the results of questionnaire surveys or personality assessment research will be influenced by the subjective factors of the subjects to some extent. Applying the discourses of historical celebrities in official history as the material of this analysis not only guarantees the reliability of the research data but also eliminates the reprocessing bias caused by the reporting of others. This article adopts the research methods of text analysis systems, which can conduct a longitudinal study of groups with high efficiency and low cost, and it can provide quantitative support for the previous qualitative research. This is the first time that a text analysis system has been applied to classical Chinese by using classical Chinese big data to study the psycho-linguistic changes of Henan celebrities, thereby providing new insights for humanities research.

## Research Methods

### Research Object

#### Selection of Years

The historical celebrities specifically refer to ancient historical celebrities, that is, the historical celebrities born between the Spring and Autumn Period and the Warring States Period and the year 1820. The year 1840 (the Opium War) was the demarcation point of modern Chinese history. Choosing the year 1820 ensured that the figures of childhood occurred in the ancient period (20 years of growth).

#### Celebrity Standard

A historical celebrity, as the name implies, means a person of historical renown. Cailie Zhang believes that the historical celebrities are “historical figures who cut a figure in a certain field in history, played a significant role in the country, the nation and the people in a certain aspect, and had a far-reaching impact on future generations.” He has also pointed out that this category of historical figures includes “immortal” positive historical celebrities and “notorious” historical celebrities (Zhang, 1994).

### Data Collection

#### Selection of Historical Celebrities

There were three sources used to identify the historical celebrities: (1) In the Dictionary of Henan Province (Shao, 1991) published by Xinhua Publishing House, edited by Wenjie Shao, 143 people from Henan Province were born in 1820. (2) “Celebrities in Henan History” (Sang, 2005), shows a total of 149 people from Henan; 3. Research on Resource Disputes of Historical Celebrities in Henan province—Dongsheng Fan (Fan, 2012) shows a total of 127 people.

“The Great Dictionary of Henan,” edited by Shao Wenjie and published by Xinhua Publishing House, is a comprehensive review of the geography, agriculture, forestry and animal husbandry, societies, dialects, ancient and modern people, and other aspects of the history and encyclopedia reference books of Henan Province. Adhering to the viewpoint of dialectical materialism and historical materialism, it factually records the history and the present in all aspects and strives to be scientific and informative. The famous people in Henan recorded in it are all the influential figures in the past dynasties. “Celebrities in Past Dynasties of Henan,” edited by Sang Jinke and published by Wuzhou Communication Press, selected nearly 200 representative famous figures in the past dynasties of Henan. Many of the historical figures evaluated in this book made brilliant and immortal achievements in their times. “Research on Resources Disputes of Historical Celebrities in Henan” by Fan Dongsheng, led to a relatively comprehensive collection of historical celebrities in Henan from the Pre-Qin Dynasty to the Qing Dynasty based on the previous studies. All three of these reference source records have a certain influence in their own time and in the field of historical figures; thus, considering the influence and achievement of these three books, this article selected historical figures that appeared in at least two of the three sources to improve the recognition and consistency of Henan historical celebrities selected for this study.

First selection: Among the three studies, 122 celebrities were selected according to their date of birth being between the Spring and Autumn Period and the Warring States Period and the year 1820 and whether they appeared in two or more of the materials.

Elimination: Considering the validity of the data, characters with fewer than 300 words in ancient texts, and dynasties with only one celebrity were excluded (this study focuses on analyzing the psycho-linguistic of groups). After elimination, there were 47 people included in the total. The specific celebrity screening data is shown in Table 1.

**Table 1:** Data presentation of the selection process of historical celebrities (refer to Appendixes I, II in Supplementary Material for the detailed list).

**Number of people appearing in two or more types of literature**	**The total number of words <300**	**Only one person in the dynasty**	**Final choice**
122	72	3	47

*[Final selection] = [the number of people appearing in the two kinds of literature]–[the total number of words <300]–[Only one person in the dynasty]*.

Regarding the distribution of historical celebrities across dynasties, there were 47 historical celebrities in the Central Plains, as shown in Table 2, including 11 in the Spring-Autumn Period and the Warring States Period (23.4%), 11 in the Han Dynasty (23.4%), 11 in the Three Kingdoms and Jin and Southern and Northern Dynasties (23.4%), 11 in the Tang Dynasty (23.4%), and three in the Song Dynasty (6.4%).

**Table 2:** The dynasties distribution of the historical celebrities in Henan.

**Dynasty**	**Number**	**Capital**	**Reign**
Spring-Autumn Period and Warring States Period	11	Luoyang	550
Han	11	Chang 'an, Luoyang	405
• Three Kingdoms and Jin and Southern and Northern Dynasties	11	• Luoyang, Chengdu, Nanjing	386
• Tang	11	• Luoyang	289
• Song	3	• Kaifeng, Luoyang, Nanjing, Beijing	319

*For simplicity and clarity, the names of capitals are based on their current names*.

All 47 Henan historical celebrities with self-expression data were male.

Among these 47 historical celebrities, except for four military generals (Yi Feng, Rengui Liu, Jian Wang, and Fei Yue), who had no academic records and one historical celebrity (Pu Zhao) who failed in imperial examinations, the remaining 42 (89.36%) historical celebrities were well-educated and had some achievements in certain fields. Therefore, most of the 47 historical celebrities were well-educated in society at that time. Even celebrities who have no relevant records still developed their advantages in other areas (military, political, etc.).

The backgrounds of the 47 celebrities are mainly based on the occupations of their fathers, as shown in Table 3. In ancient times, people were divided into four classes: officials, farmers, workers, and merchants. It can be seen from the above data that the largest proportion of historical celebrities were scholars, while historical celebrities whose fathers had no reputation status are the second largest group. If their background depends on whether their fathers are official or not, the proportion of officials is 48.94%, and the proportion of non-officials is 51.06%, which is close to 1:1. During the Spring and Autumn Period, there were 11 historical celebrities in Henan, four of whom had fathers with corresponding official positions, and the proportion of non-official fathers was 63.64%, which was higher than the overall level.

**Table 3:** Distribution of family backgrounds of historical celebrities in Henan.

**Categories**	**Number**	**Proportion %**
Scholar	23	48.94
Without fame or status	20	42.55
Farmer	3	6.38
Merchant	1	2.13

*The “Without fame or status” category in this study includes the historical celebrities who are clearly stated in the historical data and have no relevant family history*.

In terms of age, the texts were recorded when they were 30–90 years old. There were civil officials and generals. Geographically, the sample includes figures from 16 cities in Henan Province. From the perspective of the social status of the father, the sample includes both the historical celebrities whose father was a scholar and those whose father was not. On the whole, the 47 historical celebrities cover a wide range and are historical celebrities recognized by more than two kinds of materials; thus, they are representative to a certain extent.

#### Classical Chinese Data Filtering

The texts as shown in Table 4 needed for this analysis were mainly from Twenty-Five Histories and The Epistle of Chinese Celebrities (Ren et al., 2000).

**Table 4:** Textual statistics in dynasty subrogation: (refer to Appendix III in Supplementary Material for the human dimension data).

**The serial number**	**Dynasty**	**The number of people**	**TCC**	**TWC**	**LWC**	**LCR %**
1	1	11	108951	85759	67288	78.46
2	2	11	20966	15431	11650	75.50
3	3	11	8024	5973	4388	73.46
4	4	11	22402	16388	12354	75.38
5	5	3	8191	5970	4516	75.64
合计		47	168534	129521	100196	77.36

*(1) Spring-Autumn Period and the Warring States Period; (2) Han Dynasty; (3) The Three Kingdoms and Jin and Southern and Northern Dynasties; (4) Tang Dynasty; and (5) Song Dynasty*.

*TCC is the number of words in the article (excluding punctuation marks), TWC is the total number of words in the article after word segmentation, LWC is the number of LIWC words included in the article, and LCR is the LIWC coverage rate of the article (namely, the number of LIWC words divided by the total number of words in this article)*.

The Twenty-Five Histories is the general name of the 25 biographical history books on the Chinese dynasties. Each book in The Twenty-Five Histories was originally independent, and the close continuity and inheritance among them make up an oversized official history of general history. The records of social changes and human activities of the past 5,000 years are an indispensable set of historical data for the study of ancient Chinese culture. Historians, semiofficial or private historians often deliberately conceal or distort the historical facts and become the tools of the ruling class. On the whole, however, the records in The Twenty-Five Histories are generally correct in terms of historical facts and dates. It is an important basis for the study of Chinese history and has higher historical significance than other historical books (Cai, 1962). The Great Series of Letters of Famous People in the Chinese Ages is a collection of letters of famous people in the Chinese Ages. The book is arranged basically in chronological order and divided into volumes of Qin-Han-Wei-Jin, Sui, Tang, Song, Jin, Yuan, Ming, Qing, and Late Qing.

The research text is the self-statement of the research object extracted from the above two sources, including self-narrative discourse (self-expression), memorials, and letters. A text storage mode is a single person separate storage; that is, all the words of each person are organized in a text file.

If it is only an objective record of a person, it cannot be used as a self-reported discourse. The content of self-reported discourses includes the following contents: self-narrated discourse (self-expression), memorials, and letters. To ensure the authenticity of the material, we decided not to use ode and homemade prefaces because there were too many rhetorical devices.

### Research Method

The CC-LIWC dictionary is based on the Simplified Chinese LIWC Dictionary (2015 edition, SC-LIWC). The CC-LIWC dictionary contains 37 types of process words, mainly consisting of linguistic, mental, personal concern, and informal language process words. Mental process words include emotion, social, cognitive, perceptual, biological, drive, and relativity words. The classical Chinese word segmentation system uses a large-scale ancient Chinese corpus to build an ancient Chinese word segmentation dictionary through big data related technology and applies positive maximum matching to achieve classical Chinese word segmentation.

Formula 1:


$$Fr\;=\;\frac{C}{TWC}$$

In Formula 1, Fr stands for word frequency, C represents the number of words of a certain LIWC part of speech in a certain celebrity text data, and TWC represents the total number of words in a certain celebrity text data.

Based on the storage of single-person text data, this study used the CC-LIWC psycholinguistic dictionary and the classical Chinese words segmentation system to segment and counted the 37 types of mental processes word and obtained the word frequencies of 37 types of mental processes word of each historical celebrity. The word frequency data of 47^*^37 was finally obtained. After grouping words according to the five dynasties (historical dynasties), word frequencies of various parts of texts with dynasties as the group were obtained. The whole word frequency statistics were obtained through Python language coding. The operating environment and version for this article are Python 3.6.3.

This study mainly studied whether there is a difference in psychological word frequency among different dynasties; thus, the difference test method adopts a one-way ANOVA and *post-test* in this study.

## Results

In this study, 37 kinds of mental process words are analyzed by one-way ANOVA. The results show that 33 kinds of mental process words have no difference, and four kinds of mental process words have differences. The results show only the parts with significant differences.

Table 5 shows that in Henan historical celebrities, the frequency of male words (*F* = 2.938, *p* < 0. 05), the frequency of differ words (*F* = 4.767, *p* < 0. 01), the frequency of motion words (*F* = 4.042, *p* < 0) 01), and the frequency of time words (*F* = 5.412, *p* < 0.01) have significant differences among the five dynasties. Examples of each speech class are shown in Table 6.

**Table 5:** Significant differences results of one-way ANOVA of Henan historical celebrities among different dynasties.

		**Quadratic sum**	***df***	**Mean squared**	***F***	**Significance**
Male	Between the groups	0.001	4	0.000	2.938	0.031^*^
	Ingroup	0.004	42	0.000		
	Total	0.006	46			
Differ	Between the groups	0.002	4	0.001	4.767	0.003^**^
	Ingroup	0.005	42	0.000		
	Total	0.007	46			
Motion	Between the groups	0.002	4	0.000	4.042	0.007^**^
	Ingroup	0.005	42	0.000		
	Total	0.007	46			
Time	Between the groups	0.002	4	0.000	5.412	0.001^**^
	Ingroup	0.003	42	0.000		
	Total	0.005	46			

* *p < 0.05*,

** *p < 0.01*.

*Male words are mainly part of speech representing men in ancient China and belong to social process words in CC-LIWC. Differ words are mainly part of speech representing the difference in ancient China, which belong to different process words in CC-LIWC. Motion words are mainly part of speech representing movement in ancient China, while time words are mainly part of speech representing time in ancient China. Both motion words and time words belong to relative words in CC-LIWC*.

**Table 6:** Examples of four types of different words.

**Male**	**Differ**	**Motion**	**Space**
Zi	But	Walk	World
Fu	No	Up	Qin
JI	Without	Go	Without
Prince	Can't	Fight	Country
Uncle	Exhaust	Come	Road

*Since there are many words in each part of speech, only five words are shown in this study as examples to facilitate the understanding of readers*.

**Figure 1:**
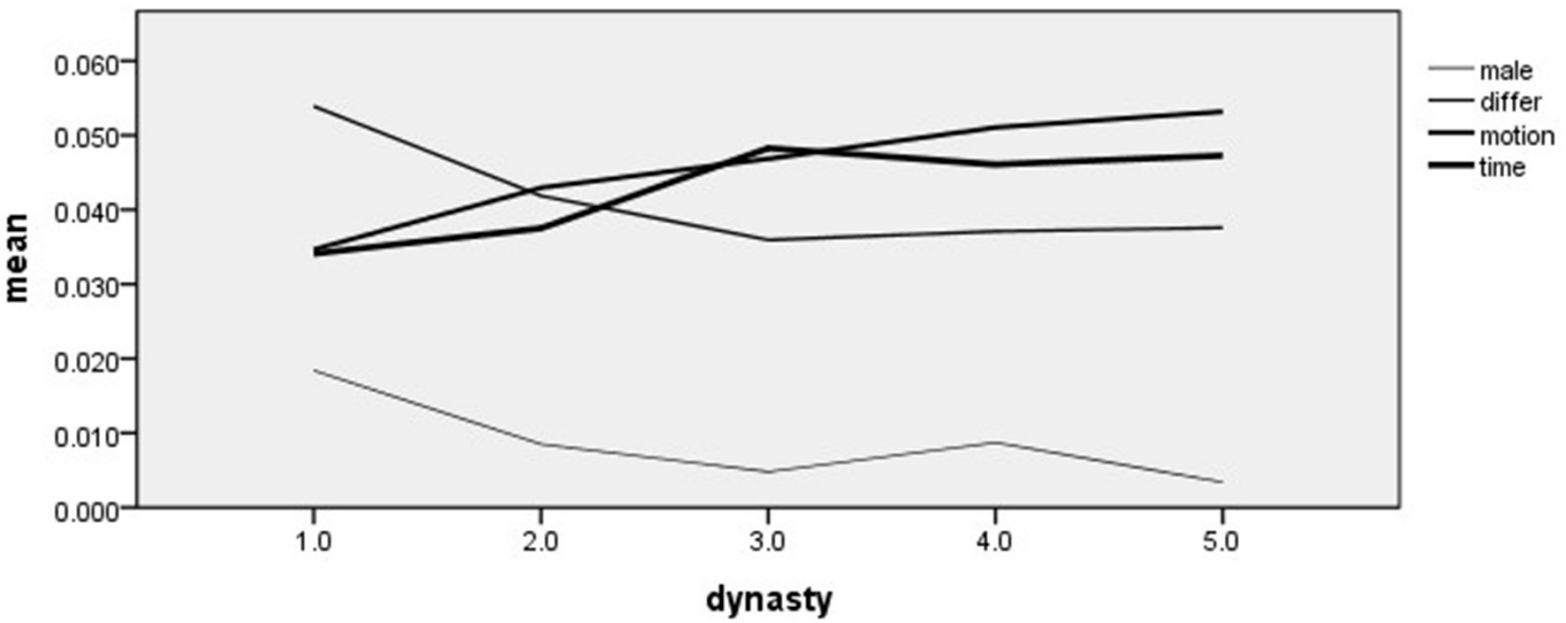
Significant different word frequency change trend of Henan historical celebrities in different dynasties. (1) Spring-Autumn Period and the Warring States Period; (2) Han Dynasty; (3) The Three Kingdoms and Jin and Southern and Northern Dynasties; (4) Tang Dynasty; and (5) Song Dynasty.

Table 7 and Figure 1 show that the male word frequency in the Spring and Autumn and the Warring States periods is significantly higher than that of the other four dynasties. In the Spring and Autumn Period and the Warring States Period, “zi” and “fu” accounted for 44.6 and 26.82% of male words, respectively, accounting for 71.44% in total. Among male words, “zi” accounted for the highest proportion. After the Spring and Autumn Period and the Warring States Period, male word frequency was in a relatively stable state.

**Table 7:** The post-test of the significant difference words frequency among different dynasties. Refer to Appendixes IV in Supplementary Material for the detailed data.

**Dependent variable**	**(i) Dynasties**	**(J) Dynasties**	**Average difference (I-J)**	**Significance**
Male	1.0	2.0	0.00989	0.029^*^
		3.0	0.01359	0.003^**^
		4.0	0.00967	0.033^*^
		5.0	0.01500	0.030^*^
Differ	1.0	2.0	0.01204	0.014^*^
		3.0	0.01799	0.000^**^
		4.0	0.01682	0.001^**^
		5.0	0.01635	0.027^*^
Motion	1.0	2.0	−0.00828	0.077
		3.0	−0.01216	0.011^*^
		4.0	−0.01639	0.001^**^
		5.0	−0.01850	0.011^*^
Time	1.0	2.0	−0.00341	0.359
		3.0	−0.01417	0.000^**^
		4.0	−0.01198	0.002^**^
		5.0	−0.01317	0.024^*^

* *p < 0.05*,

** *p < 0.01*.

Table 7 and Figure 1 show that the frequency of differ words in the Spring and Autumn Period and the Warring States Period was significantly higher than that of the other four dynasties. During the Spring and Autumn Period and the Warring States Period, the word with the highest frequency of differ words was “but,” which appeared 2,758 times (4,752 times of differ words), occupying 58% of the total differ words. According to Yan Liming, “Conjunctions Expressing Contrast _ but,” the main grammatical meaning of “but” is not to express transition but to express contrast (Yan, 2009). In the CC-LIWC dictionary, differ words belong to cognitive process words, which are mainly used to express the differences among different things, situations, and motions. The use of cognitive words reflects the active exploration and understanding of meaning. Differ words are also called exclusive words. When people try to distinguish between things in one category and things not in one category, they use exclusive words. Exclusive words, such as “rather,” “But,” “except,” and “however” are used to measure the difference between different concepts and indicate greater cognitive complexity (Newman et al., 2003). Therefore, the results of this research can show that the differential cognition of historical celebrities in Henan during the Spring and Autumn and the Warring States period was significantly higher than that of other dynasties.

Table 7 and Figure 1 show that from the Spring and Autumn and the Warring States period to the Song Dynasty, the frequency of motion words continued to increase. The motion words frequency had no significant difference between the Spring and Autumn and the Warring States period and the Han Dynasty. The frequency of motion words during the Three Kingdoms and Jin and Southern and Northern Dynasties, Tang Dynasty, and Song Dynasty is significantly higher than that in the Spring and Autumn and the Warring States period. The frequency of time words has been increasing from the Spring and Autumn and the Warring States to the Three Kingdoms and Jin and Southern and Northern Dynasties, while it was relatively stable from the Three Kingdoms and Jin and Southern and Northern Dynasties to the Song Dynasty. There is no significant difference in time words frequency between the Spring and Autumn and the Warring States Period and the Han Dynasty. The time words in the Spring and Autumn and the Warring States Period are significantly lower than those in the Three Kingdoms and Jin and Southern and Northern Dynasties, Tang Dynasty, and Song Dynasty, and the frequency of time words in Han is significantly lower than those in the Three Kingdoms and Jin and Southern and Northern Dynasties, and Tang Dynasty. Motion, exclusion, and sense words all indicate the degree to which an individual elaborated on the description of the scenario (Zhou et al., 2004). In CC-LIWC, motion words and time words are relative words, mainly used to express the relativity of things and time. Time words can also be applied to describe scenes in detail. From the changes in the word frequencies of time words and motion words in different dynasties, it can be concluded that the relative words of the historical celebrities in Henan province are constantly increasing.

## Discussion

Group psychology, as the expression of group cognition, emotion, and expectations of social life, is a direct reaction of people to social structure and social operation and other social development. It reflects the thoughts, feelings, and expectations of people of current and future social life. It is not only the “weathervane” of social development but also the “barometer” of social reality (Yu and Wang, 2017).

### During the Spring and Autumn and the Warring States Period, 100 Schools of Thought Contended, and the Status of “Scholars” in Henan Rose, With Significant Cognitive Differences

During the Spring and Autumn and the Warring States period, agricultural production developed dramatically, and the improvement of social productivity was mainly due to the emergence and application of ironware and cattle farming. As the position of agricultural production became impregnable, the male was thus “superior” and became the dominant leader and manager of the clans (Zhou, 2004). During the Spring-Autumn Period and the Warring States Period, with the further development of productive forces, it became possible for some people to specialize in knowledge production. The rapid change of society, the decline of royal power, the difficulty in running official schools, and the rise of private schools promoted the formation of scholarly classes (Shi, 2012). Xiu He noted in KungYang Chuan• The First Year of Cheng Gong that “The interpretation of scholar, farmer, artisan, and merchant specifies that scholar is the one whose own virtue is commensurate with one's social status.” Xuan Zheng noted in the Book of Rites • Book of Learning that “A scholar is an intellectual.” Zhao Wei noted in National Language • Qi Language that “A scholar is a person who teaches and preaches.” Those people who, in the old days, were thought to have moral character and extensive knowledge and ability are now commonly referred to by scholars as “intellectuals.” During the Spring-Autumn Period and the Warring States Period, scholars had a wide range of meanings, but, in the Spring-Autumn Period and the Warring States Period and later times, the definition of “scholars” was already intellectuals (Miao, 2007). In the Spring-Autumn Period and the Warring States Period, the male word that accounted for the highest proportion was “Zi.” The word “Zi” in ancient Chinese is mainly interpreted as a respectful title for people in ancient times, such as teachers or moral, and learned people, such as scholars in the pre-Qin period (from the interpretation of Chinese Dictionary). During the Spring and Autumn Period and the Warring States Period, the high proportion of “Zi” (compared with the Tang Dynasty, whose capital was also in Luoyang, the proportion of “Zi” was still significantly higher) proves the rise of the social status of scholars in Henan to some extent.

During the Spring and Autumn Period and the Warring States Period, the Central Plains was undoubtedly the most important political, economic, and cultural center area. At the same time, the high freedom of politics and ideology in the Spring and Autumn Period and the Warring States Period gave rise to many ideological masters. The Spring and Autumn and the Warring States Period was the period of city creation. At that time, the changes were intense, and creation, communication, and development were dominant. Therefore, the relatively loose political environment created the brilliant period of the pre-Qin scholars and contention of a hundred schools of thought (Song, 2000). In the history of Chinese culture, the Spring and Autumn and the Warring States Period is undoubtedly a vibrant era. In the further 2,000 years, regardless of culture or thought, the glory of that time was never surpassed. Scholars in the Spring and Autumn Period and the Warring States Period regarded “Tao” as the main knowledge they preached, but the “Tao” they said was very different in connotation. The “benevolence” of Confucius, “love each other” and “mutual benefit” of Mozi, “rule of law” of the legalist, and different perspectives from other scholars offered suggestions for solving the common problems of the times (Yang, 2001). The academic situation in which hundreds of scholars contended and argued with each other was an unprecedentedly grand occasion and occupies an important position in the history of Chinese cultural development.

The social economy is the basis of social development. The economic and social development in the Spring and Autumn Period and the Warring States Period made people no longer limited to the pursuit of food and clothing and put forward new requirements for culture, ideology, systems, and management. As the saying goes, “We know propriety when we have enough granaries; we know honor and disgrace when we have enough food and clothing.” According to the hierarchy of needs theory by Maslow, the needs of people in the Spring and Autumn and the Warring States periods were no longer focused on the physiological and safety needs but more on self-realization needs. At the same time, the social and political needs and the guidance of social culture eventually led to the change of social class and the psycho-linguistic change of social group, which promoted the rise of the “scholar” class and the high difference cognition in the Spring and Autumn Period and the Warring States Period. According to the records, the Spring and Autumn Period and the Warring States Period were the most developed periods of Central Plains culture (Han, 2012b). Due to the political needs of various countries, the culture of the Central Plains witnessed unprecedented development during this period.

### From “The Contention of a Hundred Schools of Thought” to “The Supremacy of Confucianism,” Scholars in Henan Were in Decline, and Their Cognitive Exclusiveness Was Reduced Since the Han Dynasty

After the Han Dynasty, the demand for social politics and diversity of social culture decreased, and Confucianism was chosen to guide and manage society, which led to the decline of the status of scholars, the decline of cultural diversity, and the unity of thoughts of the people. From “The Contention of a Hundred Schools of Thought” to “The Supremacy of Confucianism,” that is, the evolution from pre-Qin scholars to Han Confucian classics. During this evolution, scholars from multiple schools gradually declined. The root is that the ruling class no longer needed so many theories to guide its expansion in the situation of great unification. Moreover, the prosperity of theories will threaten the stability of its regime. Strictly controlling the thoughts of people and maintaining social stability were what they desired, and the decline of studies of scholars became an inevitable trend. The “contending of a hundred schools of thought” in the Spring and Autumn Period and the Warring States was destroyed by the Han Dynasty, and only Confucianism maintained its predominant momentum (Gong, 2011). The historical celebrities in Henan were political courtiers at that time, and their text analysis shows that after the Spring and Autumn Period and the Warring States Period, the frequency of male words and different words (exclusive words) showed clear downward trends.

The Three Kingdoms and Jin and Southern and Northern Dynasties were periods of national division and national integration. During this period, regimes and territories changed and were exchanged. In terms of ideology and culture, the Supremacy of Confucianism was broken, metaphysics became popular, Buddhism and Taoism developed, and the ideas of “three religions homologous” and “three religions merge” appeared. The different degrees of the revival of the pre-Qin theory led to the emancipation of the mind and the integration of nationalities into Chinese history. National development is characterized by diversity and unity. This period experienced national wars, friendly exchanges, population migration, government reforms, and strengthened management. The national economy developed, national cultures had a symbiosis, and humans and nature lived in harmony (Liu, 2006). Civilizations blended and developed. As a result, during this period, the exclusive cognition of Henan historical celebrities further decreased, while the relative cognition further improved.

### During the Period of the Three Kingdoms and Jin and Southern and Northern Dynasties, Political Powers, and Territories Changed, and Historical Celebrities in Henan Showed More Time and Space Relativity

The Three Kingdoms and Jin and Southern and Northern Dynasties were the most frequent periods of regime change in Chinese history, leading to the need for contemporary, and future generations to use more time words to express the accuracy of time. Therefore, the frequency of time words used by Henan historical celebrities reached the highest value during the Three Kingdoms and Jin and Southern and Northern Dynasties, and there was a significant difference in the frequency of time words compared to the Spring and Autumn and the Warring States period and the Han Dynasty. Afterward, with the slow change of regimes, the use of time words was relatively stable. There was no significant difference in the frequency of using time words among Tang, Song, and the Three Kingdoms and Jin and Southern and Northern Dynasties.

Xuewei Zhai believes that the contribution of native psychology is to advocate human psychology and to advocate sociality rather than biology, and localization is the socialization or culture of psychology after all (Wu, 1997). The rise of the class of “scholars” during both the Spring and Autumn Period and the Warring States Period had a high and significant difference in the cognition of difference, and the beginning of the decline of the cognition of difference in the Han Dynasty and the significant increase of motion words and time words in The Three Kingdoms and Jin and Southern and Northern Dynasties. All these findings are highly consistent with the development of social history. The guidance of social politics and the cultural environment has an important influence on the psycho-linguistic changes of social class. These psycho-linguistic changes reflect social class changes and cognitive psychological changes. The historical celebrities studied in this study were scholars, who were more easily guided by social politics and social culture. In the future, decision-makers and managers will still be faced with the problem of correctly guiding groups under different social situations. Political needs at that time, which help to create a corresponding social atmosphere and guide social groups with the help of the social system, social culture, and social opinion, may be the topics that we should pay attention to.

Please refer to Appendix V in Supplementary Material for the annotations of proper Chinese nouns in the discussion section.

## Limitations

The research on historical celebrities has the following limitations. (1) There is a lack of comparative studies on the historical celebrities in other regions, of psychological research of modern Henan historical celebrities, and of a horizontal and vertical understanding of the historical celebrities. (2) This article currently only reveals the psycho-linguistic changes of historical celebrities in Henan from text analysis and word frequency statistics. A textual personality analysis can be further added to enrich and verify the current research results. (3) Due to the fixed writing format of classical Chinese, function words in classical Chinese were applied very frequently, and most of the same words appeared repeatedly, resulting in a large proportion of function words in the statistical results. However, for classical function words, there is currently no more suitable way of interpretation in its psychological sense. (4) In classical Chinese, polysemous and polysemous words are common, which has a certain influence on the classification of words in the dictionary. This will also affect the accuracy of the final analysis results. (5) Limited by the influence of traditional culture, especially the “Supremacy of Confucianism” after the Spring and Autumn and the Warring States period, few females could leave footprints in official historical data in history. Thus, this article lacks research on female celebrities.

## Conclusion

The Chinese civilization has a long history, and the Central Plains, with Henan as the core, is one of its birthplaces. Understanding the psychological changes in Henan is of great significance for understanding the evolution and formation of national cultural psychology. Using classical Chinese big data, this article took the self-reported discourses of historical celebrities in Henan in official history and researched the psycho-linguistic changes of historical celebrities in Henan based on the classical Chinese LIWC psycholinguistic dictionary and the classical Chinese word segmentation system. The results showed that the frequency of male words (*F* = 2.938, *p* < 0.05), the frequency of differ words (*F* = 4.767, *p* < 0. 01), the frequency of motion words (*F* = 4.042, *p* < 0. 01), and the frequency of time words (*F* = 5.412, *p* < 0.01) used by Henan historical celebrities showed significant differences among the five dynasties. Among them, the frequency of male words and different words showed declining trends, while the frequency of motion words and time words showed an increasing trend. The changing trends of these four types of word frequencies show that the Spring and Autumn and the Warring States periods, Han, and The Three Kingdoms and Jin and Southern and Northern Dynasties were the psycho-linguistic turning points of historical celebrities in Henan.

As the historical celebrities have been deceased for a long time, results from questionnaires or personality assessments will be affected to some extent by subjective factors. Using the self-reported discourses of historical figures in official history as the units of analysis not only ensures the reliability of the research data but also eliminates the reprocessing bias caused by the narratives of others, such as biographies. This study adopts a text analysis system as the research method, which can conduct a single longitudinal study on the population, with high efficiency and low cost, and can provide quantitative support for previous qualitative research. This is the first time that a text analysis system has been applied to classical Chinese by using ancient literature to study the psycho-linguistic changes of Henan celebrities, thereby providing new insights for humanities research. This study still has limitations. In the follow-up, we will continue to strengthen the classification accuracy of the CC-LIWC dictionary and at the same time increase the cross-sectional and longitudinal comparative studies of the historical celebrities, including psycho-linguistic changes of female celebrities and in-depth textual personality analysis.

## Data Availability Statement

The raw data supporting the conclusions of this article will be made available by the authors, without undue reservation.

## Author Contributions

YZ is responsible for the whole research process of this paper, including data collection, data processing, research results, discussion, etc. MF is mainly responsible for the research and verification of CC_LIWC dictionary, while FX is mainly responsible for the research of classical Chinese words segmentation system. Thanks a lot for Teacher HL guidance on cultural research, as well as Teacher TZ guidance on article conception, writing, and the whole research process. All authors contributed to the article and approved the submitted version.

## Conflict of Interest

The authors declare that the research was conducted in the absence of any commercial or financial relationships that could be construed as a potential conflict of interest.

## Publisher's Note

All claims expressed in this article are solely those of the authors and do not necessarily represent those of their affiliated organizations, or those of the publisher, the editors and the reviewers. Any product that may be evaluated in this article, or claim that may be made by its manufacturer, is not guaranteed or endorsed by the publisher.
