# Supplementary material for: Psycho-Linguistic Changes Associated With Historical Celebrities in Henan Using Classical Chinese Big Data
Source: Front Psychol. 2021 Sep 14;12:648677. doi: 10.3389/fpsyg.2021.648677 (PMC8476835; doi:10.3389/fpsyg.2021.648677)
Supplement: Supplementary file 1 [file Data_Sheet_1.PDF]

Supplementary Material

1 Appendix I Details of the selection process of historical celebrities

The serial number	Birth history	Names of historical celebrities	The number of occurrences among the three choice sources	Total words of text (TCC)	The total number of words in the text <300 will be deleted	Only one in dynasty was removed	Final choice
1	1	Lao tze	2	8735			1
2	1	Zi gong	2	952			1
3	1	Mozi	2	11899			1
4	1	Fan li	3	629			1
5	1	Shu in	3	7309			1
6	1	Zhang Yi	2	5039			1
7	1	Zi chan	2	2416			1
8	1	Zhuangzi	3	3057			1
9	1	Lv buwei	3	544			1
10	1	Han fei	3	67701			1
11	1	Shang Yang	3	670			1
12	2	Chen ping	3	593			1
13	2	Zhang liang	2	993			1
14	2	ChaoCuo	3	4310			1
15	2	Zhang shizhi	3	315			1
16	2	Jia yi	3	7335			1
17	2	Feng yi	3	443			1
18	2	Yuan shao	3	1340			1
19	2	Xun you	2	334			1
20	2	Xun yu	3	1317			1
21	2	Cai yong	3	2928			1
22	2	Zhang heng	3	1058			1
23	3	Sima yi	3	1041			1
24	3	Deng ai	3	954			1
25	3	Ruan ji	3	1034			1
26	3	Zhong yao	3	394			1
27	3	Wang jun	2	442			1
28	3	Xie xuan	2	785			1
29	3	Pan yue	3	440			1
30	3	Xie lingyun	3	310			1
31	3	Fan ye	3	377			1
32	3	Fan zhen	3	1291			1
33	3	Jiang yan	3	956			1

34	4	Yao chong	3	992			1
35	4	Liu rengui	2	360			1
36	4	Wu jing	2	1380			1
37	4	Zhang xun	2	396			1
38	4	Zhang shuo	2	1021			1
39	4	Bai juyi	2	10554			1
40	4	Yuan jie	3	1328			1
41	4	Han yu	3	3059			1
42	4	Liu yuxi	3	302			1
43	4	Wang jian	2	397			1
44	4	Yuan zhen	3	2613			1
45	5	Zhao pu	3	852			1
46	5	Sima guang	2	5498			1
47	5	Yue fei	3	1841			1
48	0	Fuxi	2	0	1		
49	0	Huangdi	2	0	1		
50	0	Yiyi	2	0	1		
51	0	Bigan	3	0	1		
52	0	Jiangshang	2	0	1		
53	1	Shen zhuliang	2	0	1		
54	1	Hui shi	2	143	1		
55	1	Baili xi	2	0	1		
56	1	Sunshu ao	3	88	1		
57	1	Wei liao	2	0	1		
58	1	Shen buhai	3	0	1		
59	QIN	Chen sheng	3	177	1		
60	QIN	Wu guang	3	0	1		
61	2	Li shiqi	2	0	1		
62	2	Guan ying	3	0	1		
63	2	Zhang cang	3	0	1		
64	2	Sang hongyang	3	0	1		
65	2	Heng kuan	2	0	1		
66	2	Yao qi	2	93	1		
67	2	Deng yu	2	245	1		
68	2	Chen fan	3	0	1		
69	2	Yuan shao	3	215	1		
70	2	Guo jia	3	0	1		
71	2	Xun yue	2	0	1		
72	2	Xu shen	3	0	1		
73	2	Cai wenji	3	0	1		
74	2	Du shi	3	0	1		
75	2	Zhang zhongjing	2	0	1		
76	2	Li ying	2	0	1		
77	2	Chen yu	2	0	1		
78	3	Huang zhong	2	0	1		

79	3	Chen qun	2	0	1		
80	3	He yan	2	0	1		
81	3	Wang bi	2	0	1		
82	3	Xie an	3	196	1		
83	3	Xiang xiu	2	292	1		
84	3	Guo xiang	2	0	1		
85	3	Gan bao	3	0	1		
86	3	Xie daoyun	2	0	1		
87	3	Zhong rong	3	203	1		
88	3	Yu xin	3	0	1		
89	SUI	Han qinhu	3	114	1		
90	SUI	Zhai rang	3	0	1		
91	4	Xuan zang	3	0	1		
92	4	Li yanshou	3	0	1		
93	4	Shangguan yi	3	62	1		
94	4	Shangguan waner	2	0	1		
95	4	Liu xiyi	2	0	1		
96	4	Cen sen	2	0	1		
97	4	Du shenyan	2	99	1		
98	4	Cui hao	3	0	1		
99	4	Du fu	3	0	1		
100	4	Li he	3	0	1		
101	4	Li shangyin	3	0	1		
102	4	Yi hang	2	23	1		
103	4	Wu daozi	3	0	1		
104	5	Shi shouxin	2	67	1		
105	5	Lv mengzheng	3	290	1		
106	5	Niu gao	3	56	1		
107	5	Chen tuan	2	188	1		
108	5	Shao yong	3	22	1		
109	5	He zhu	3	0	1		
110	5	Li jie	3	0	1		
111	5	Zhu dunru	2	0	1		
112	MING	Wang tingxiang	3	247	1		
113	MING	Zhu zaiyu	2	0	1		
114	MING	Wang duo	3	0	1		
115	QING	Zhang boxing	2	0	1		
116	QING	Tang bin	2	0	1		
117	QING	Hou fangyu	2	0	1		
118	QING	Wu qijun	2	0	1		
119	QING	Li wencheng	2	0	1		
120	QIN	Li si	3	3100		1	
121	YUAN	Xu heng	3	3894		1	
122	MING	Shi kefa	3	1101		1	

Note: In the dynasty column: 0= Before the Spring and Autumn Period and the Warring States Period;1=Spring-Autumn Period and Warring States Period;2= Han Dynasty;3= The Three Kingdoms and Jin and Southern and Northern Dynasties;4= Tang Dynasty;5= Song Dynasty

Note: "The total word of the text is less than 300 and excluded"; Fill 1 in the column to indicate that the corresponding person is excluded because the total word of the text is less than 300;

"Only one person in the dynasty was removed", filling 1 means that after the deletion of <300 words, only one person in the dynasty was removed;

Fill in 1 for "final selection" to indicate that the person was finally selected as a study subject.

There was no repetition of celebrities between dynasties.

2 Appendix II List of 47 Henan Historical Celebrities:

The serial number	Birth history	Names of historical celebrities	Introduce	Gender	Date of birth
1	1	Lao Tzu	the founder of the Taoist school. born in Kuxian county of Chu (today's Luyi, Henan) during the Spring and Autumn Period	Male	Unknown
2	1	Zi Gong	the ancestor of Chinese Confucian merchants, born in Wei (present-day Jun County, Henan)	Male	BC520-Unknown
3	1	Mozi	Mozi, an outstanding thinker, educator and founder of mohist school, born in Lushan, Henan province	Male	BC468-BC376
4	1	Fan li	a famous statesman, strategist and merchant in ancient China, was honored as the saint of Commerce by later generations. born in Wan of Chu State (now Nanyang, Henan Province).	Male	Unknown
5	1	Su qin	a famous strategist, born in Xuanli, Luoyang (today's Luoyang, Henan) during the Eastern Zhou Dynasty.	Male	Unknown - BC284
6	1	Zhang yi	a political strategist in the Warring States Period, born in the State of Wei (now Kaifeng, Henan province).	Male	Unknown - BC310
7	1	Zi Chan	A well-known politician, thinker, and pioneer of Legalism, Zichan, born in Zheng Guo (now Xinzheng, Henan).	Male	Unknown - BC520
8	1	Zhuangzi	Famous thinker, philosopher, Taoist school representative Zhuangzi, Meng of Song state, born in Min Quan County, Henan province. Also called ZhuangZhou.	Male	BC369-BC286

9	1	Lv buwei	A famous businessman and statesman,born in Puyang city of Wei (in present-day Yanshi county, Henan Province). His tomb is located in Yanshi City, Luoyang	Male	Unknown - BC235
10	1	Han Fei	the master of legalist thought, was a man of Korea in the late Warring States Period (now xinzheng, Henan province, has a different opinion)	Male	BC280-BC233
11	1	Shang yang	an outstanding statesman and reformer in ancient China, = born in the middle of the Warring States Period in Wei (now Neihuang, Henan)	Male	BC390-BC338
12	2	Chen ping	the founder of the Western Han Dynasty and a famous strategist, was a man of Yangwu (now Yuan Yang, Henan, has a different opinion)	Male	Unknown - BC178
13	2	Zhang liang	one of the three outstanding statesmen and strategists in the late Qin and early Han Dynasties, was a native of Baofeng, Henan province. has a different opinion.	Male	Unknown - BC185
14	2	Cao cuo	The Western Han Dynasty statesman, a native of Yingchuan (now Yuzhou, Henan), has the tomb on the west side of the Martyrs Cemetery in Xuchang.	Male	BC200-BC154
15	2	Zhang shizhi	who was famous for his impartial law enforcement, was born in Duyang (now south of Henan) in the Western Han Dynasty. There are Shizhi Zhang's Tomb and Shizhi Zhang's Temple in Fangcheng nowy.	Male	Unknown
16	2	Jia yi	an outstanding political commentator and writer in the early Western Han Dynasty, was from Luoyang.	Male	BC200-BC168
17	2	Feng yi	the founding hero of the Eastern Han Dynasty and a famous military strategist, born in Yingchuan Father City (now Baofeng, Henan), and his tomb is in Baofeng County today.	Male	Unknown -34
18	2	Yuan shao	Shu Yuan and Shao Yuan, the heroes of the late Han Dynasty, were both from Runan Ruyang (now Shangshui, Henan).	Male	Unknown -202
19	2	Xun you	Father Xun Yi (yí) is Xun Yu's uncle brother	Male	157-214
20	2	Xun yu	An outstanding politician, strategist, Cao Cao adviser Xun or, from Yingchuan Yingyin (now Xuchang, Henan).	Male	162-212

21	2	Cai yong	Famous writer and calligrapher, Liuyu people of Chen(now Qixian County, Henan Province, or Weishi County).	Male	132-192
22	2	Zhang heng	An outstanding scientist and writer in ancient times, known as the saint of science, born in Xi'e, Nanyang (now Shiqiao Town, Nanyang City, Henan)	Male	78-139
23	3	Sina yi	An outstanding statesman and military strategist in the Wei Kingdom, he founder of the Western Jin Dynasty, born in Wen County, Hanoi (now Wen County, Henan)	Male	179-251
24	3	Deng ai	an outstanding military strategist and general of the Wei Kingdom, born in Jiyang (now Xinye, Henan) in Yiyang.	Male	197-264
25	3	Ruan ji	a writer of the Wei Dynasty and one of the seven sages of the bamboo forest, born in the Wei clan of Chenliu (now Weishi, Henan). There are Ji Ruan tomb and Ji Ruan shouting platform in current Weishi	Male	210-263
26	3	Zhong yao	a famous calligrapher and politician in Cao Wei .He was the ancestor of calligraphy history and the originator of regular script,born in Yingchuan Changshe (now Changge, Henan).	Male	151-230
27	3	Wang jun	born in Hu County, Hongnong County (now Lingbao City, Henan Province). Famous general in the Western Jin Dynasty.	Male	206-285
28	3	Xie xuan	a famous general in the Eastern Jin Dynasty, born in Taikang, Henan.	Male	343-388
29	3	Pan yue	a writer and beautiful man, born in Zhongmu (now Zhongmu County, Henan).	Male	247-300
30	3	Xie lingyun	the founder of the School of Landscape Poetry in the history of Chinese literature, and the great historical traveler,born in Taikang.	Male	385-433
31	3	Fan ye	The famous historian and writer, born in Shunyang (now Xichuan, Henan) in the Southern Song Dynasty.	Male	398-445
32	3	Fan zhen	a thinker and outstanding atheist in the Southern Dynasties between Qi and Liang, was from Nanxiang Wuyin (now Biyang, Henan) (with different opinions)	Male	450-510
33	3	Jiang yan	Famous minister of Qiliang in the Southern Dynasties, famous writer, with allusions such as the brilliant writing of flowers, Jiang Lang, etc., a native of Jiyang Kaocheng (now Henan Minquan)	Male	444-505

34	4	Yao chong	a famous minister known as the prime minister at the time of rescue, was from Xiashi, Shanzhou (now Shanxian County, Henan).	Male	650-721
35	4	Liu rengui	Minister and famous military general, from Zhou Weishi (now Weishi, Henan).	Male	601-685
36	4	Wu jing	born in Bianzhou Junyi (now Kaifeng, Henan). Minister of the Tang Dynasty, a famous historian.	Male	670-749
37	4	Zhang xun	Born in Nanyang, Dengzhou in "New Tang Book". Famous minister in the middle of Tang Dynasty.	Male	709-757
38	4	Zhang shuo	Literary and politician,originally from Fanyang and later moved to Luoyang, Henan.	Male	667-730
39	4	Bai juyi	The well-known realist poet,born in Xinzheng, Henan.	Male	772-846
40	4	Yuan jie	pioneer of the ancient prose movement and writer Yuan Jie, born in Lushan, Henan.	Male	719-772
41	4	Han yu	Outstanding writer, thinker and politician, from Heyang (now Mengzhou, Henan), Hanoi (with different opinions)	Male	768-824
42	4	Liu yuxi	The famous poet, known as a poet, born in Luoyang (with different terms).	Male	772-842
43	4	Wang jian	written as Zhongchu, born in Yingchuan, Xuzhou (now Xuchang City, Henan Province), minister and poet of the Tang Dynasty.	Male	767-830
44	4	Yuan zhen	Famous poet Yuan Zhen, from Luoyang	Male	779-831
45	4	Zhao pu	The famous minister, born in Ji County, Youzhou (now Beijing), and later moved to Changshan (now Zhengding, Hebei) and Luoyang (now Luoyang, Henan).	Male	922-992
46	4	Sima guang	a politician, writer, and historian, born in Sushui Township, Xia County, Shanxi Province, and born in Guangshan County, Henan Province.	Male	1019-1086
47	4	Yue fei	a famous strategist, military strategist, and anti-gold warrior in my country’s history, born in Tangyin (now Tangyin, Henan Province) in Xiangzhou in the Northern Song	Male	1103-1142

Note:1=Spring-Autumn Period and Warring States Period;2= Han Dynasty;3= The Three Kingdoms and Jin and Southern and Northern Dynasties;4= Tang Dynasty;5= Song Dynasty

3 Appendix III Statistical Data of text Analysis of Historical Celebrities in Henan:

序号	朝代	名人姓名	TCC	TWC	LWC	LCR

1	1	Lao Tzu	8735	6820	5633	82.60%
2	1	Zi Gong	952	700	576	82.29%
3	1	Mozi	11899	9365	7663	81.83%
4	1	Fan li	629	450	346	76.89%
5	1	Su qin	7309	5644	4317	76.49%
6	1	Zhang yi	5039	3830	2885	75.33%
7	1	Zi Chan	2416	1838	1393	75.79%
8	1	Zhuangzi	3057	2388	1867	78.18%
9	1	Lv buwei	544	394	310	78.68%
10	1	Han Fei	67701	53813	41888	77.84%
11	1	Shang yang	670	517	410	79.30%
12	2	Chen ping	593	437	339	77.57%
13	2	Zhang liang	993	729	551	75.58%
14	2	Cao cuo	4310	3190	2411	75.58%
15	2	Zhang shizhi	315	224	172	76.79%
16	2	Jia yi	7335	5517	4277	77.52%
17	2	Feng yi	443	314	227	72.29%
18	2	Yuan shao	1340	934	638	68.31%
19	2	Xun you	334	257	196	76.26%
20	2	Xun yu	1317	983	766	77.92%
21	2	Cai yong	2928	2084	1507	72.31%
22	2	Zhang heng	1058	762	566	74.28%
23	3	Sina yi	1041	783	602	76.88%
24	3	Deng ai	954	720	534	74.17%
25	3	Ruan ji	1034	731	491	67.17%
26	3	Zhong yao	394	306	232	75.82%
27	3	Wang jun	442	309	210	67.96%
28	3	Xie xuan	785	577	410	71.06%
29	3	Pan yue	440	317	213	67.19%
30	3	Xie lingyun	310	232	171	73.71%
31	3	Fan ye	377	276	208	75.36%
32	3	Fan zhen	1291	1043	827	79.29%
33	3	Jiang yan	956	679	490	72.16%
34	4	Yao chong	992	749	563	75.17%
35	4	Liu rengui	360	263	197	74.90%
36	4	Wu jing	1380	971	693	71.37%
37	4	Zhang xun	396	303	242	79.87%
38	4	Zhang shuo	1021	737	515	69.88%
39	4	Bai juyi	10554	7890	6090	77.19%
40	4	Yuan jie	1328	980	722	73.67%
41	4	Han yu	3059	2128	1580	74.25%
42	4	Liu yuxi	302	206	137	66.50%
43	4	Wang jian	397	281	203	72.24%
44	4	Yuan zhen	2613	1880	1412	75.11%

45	5	Zhao pu	852	594	406	68.35%
46	5	Sima guang	5498	4028	3108	77.16%
47	5	Yue fei	1841	1348	1002	74.33%

Note: In the dynasty column: 0= Before the Spring and Autumn Period and the Warring States Period;1=Spring-Autumn Period and Warring States Period;2= Han Dynasty;3= The Three Kingdoms and Jin and Southern and Northern Dynasties;4= Tang Dynasty;5= Song Dynasty

Note: TCC is the number of words in the article (excluding punctuation marks), TWC is the total number of words in the article after word segmentation, LWC is the number of LIWC words included in the article, and LCR is the LIWC coverage rate of the article (namely, the number of LIWC words divided by the total number of words in the article).

4 Appendix IV Post-test of the Difference of Word Frequency of Historical Celebrities in Henan in Different Dynasties:

dependent variable	(I) dynasties	(J) dynasties	average difference (I-J)	Significance
male	1.0	2.0	.00989	.029*
		3.0	.01359	.003**
		4.0	.00967	.033*
		5.0	.01500	.030*
	2.0	3.0	.00370	.403
		4.0	-.00022	.960
		5.0	.00510	.450
	3.0	4.0	-.00392	.376
		5.0	.00140	.835
	4.0	5.0	.00532	.430
differ	1.0	2.0	.01204	.014*
		3.0	.01799	.000**
		4.0	.01682	.001**
		5.0	.01635	.027*
	2.0	3.0	.00594	.211
		4.0	.00477	.313
		5.0	.00430	.550
	3.0	4.0	-.00117	.804
		5.0	-.00164	.819
	4.0	5.0	-.00047	.947
motion	1.0	2.0	-.00828	.077
		3.0	-.01216	.011*
		4.0	-.01639	.001**
		5.0	-.01850	.011*
	2.0	3.0	-.00389	.399
		4.0	-.00812	.082
		5.0	-.01022	.150
	3.0	4.0	-.00423	.359
		5.0	-.00633	.368
	4.0	5.0	-.00211	.764
time	1.0	2.0	-.00341	.359
		3.0	-.01417	.000**

	4.0	-.01198	.002**
	5.0	-.01317	.024*
2.0	3.0	-.01077	.005**
	4.0	-.00857	.024*
	5.0	-.00976	.089
3.0	4.0	.00219	.554
	5.0	.00100	.859
4.0	5.0	-.00119	.833

Note：*p<0.05, **p<0.01。

5 Appendix V Notes on Chinese Proper Nouns:

Noun:	paraphrase
Schools of thought contend	The contending of a hundred schools of thought refers to the emergence of different schools of thought among intellectuals in the Spring and Autumn Period (770-476 BC) and the Warring States Period (475-221 BC), as well as the contending among various family schools.
Scholar	Generally refers to the folk talents with a certain ability, Confucius put forward the theoretical standard of scholar: as long as the person who is strict with himself, loyal to the monarch and patriotic. As a low-level aristocrat, a scholar has the right to rule the common people and the obligation to defend the country. Its embryonic stage, is the lowest in the ancient aristocratic class a group. And the lowest layer in this group is connected with the plebeian.
clan	In primitive society, the collective of people connected by blood relations, marriage was forbidden within the clan, and the collective possession of means of production, collective production and collective consumption were carried out collectively. Also called a gens commune.
KungYang Chuan• The First Year of Cheng Gong	The Warring States Period of Qi people Ram Gao Zhuan. It is a special interpretation of the Spring and Autumn Annals. Its date is the same as that of the Annals of Spring and Autumn Annals, that is, from 722 to 481 BC. The interpretation of the Annals of Spring and Autumn is very brief and focuses on elucidating the principles of Confucius' Annals of Spring and Autumn. Gongyang Zhuan is an important scripture in the study of New Text Confucianism, with strong didacticism and relatively low literary value. The scholars of New Text Confucianism often use it as a tool to discuss politics.
Book of Rites • Book of Learning	It is a treatise on education in ancient China. It is one of the books of the Book of Rites (Book of Little Dai Rites), a monobook on ancient Chinese laws and regulations. It is the earliest book in world history that specifically discusses education and teaching issues. It is generally considered to be a work of the Si Meng School in the late Warring States Period of China. According to Guo Moruo's textual research, the author is Le Zhengke. The text is concise and comprehensive, and the metaphor is vivid. It systematically and comprehensively clarifies the purpose and function of education, teachers' status and role, and the relationship between teachers and students in the process of education and the relationship between the classmate, more systematic and comprehensive summary and sums up the Chinese pre-qin period education experience.

National Language • Qi Language	Guoyu mainly records the opinions of the aristocrats from the late Western Zhou Dynasty to the Spring and Autumn Period. It portrays the characters' personalities, expresses the thoughts and fates of different characters, and records the magnificent historical events through the language with different styles and characteristics. Writing history in language is vivid and concise, which has been praised by all generations. It is the earliest national history in ancient China.
"Tao"	Tao is a noun used by the Chinese people for understanding nature. It means the running track or trajectory of everything, or the changing and moving situation of things. Because all things are not things, coincidentally, uniform adherence to a certain thing, without exception.
"love each other" and "mutual benefit",	In Mozi's thought, "loving each” refers to "other simultaneously" and "loving each other without discrimination", which means to love all people without discrimination, such as those close to each other, high or low, rich or poor. "Mutual benefit" refers to "mutual thief", which advocates that people help each other, seek welfare, and oppose competing with each other. The essence of Mozi's thought of "mutual love and mutual benefit" is a kind of flexible management, it is through the interaction between people love each other to improve interpersonal relationship, eliminate the destructive conflict, create a good social environment, make people to love, which can satisfy the interests of everyone, this accords with the need of naturalness, and conforms to the social moral laws.
"The Supremacy of Confucianism"	It was Dong Zhongshu's idea of ruling a country, which was put forward in the first year of Yuanguang (134 B. C.), and put into practice during the reign of Emperor Wudi of the Han Dynasty. It is the feudal ideological rule policy implemented by Emperor Wudi of the Western Han Dynasty, and it is also the symbol that Confucianism occupies the dominant position in Chinese culture.
The metaphysics	During the period of Wei and Jin Dynasties, the meaning of metaphysics refers to the two aspects of making speech and doing things, and most of them take making speech and doing things elegant as far as making speech and doing things elegant as far as making speech and doing things elegant. "Xuanyuan" refers to the ontological issues that are far away from the concrete things and specifically discuss the "transcendent words and unique images". The central issue of the discussion is the question of "whether there is an end or not", that is to say, the discussion on the basis of the existence of everything in heaven and earth by means of speculative method, that is to say, the discussion on the basis of the existence of everything in the form of ontology and metaphysics, which is far away from "things" and "affairs". It is the first attempt in the history of Chinese philosophy to make Chinese philosophy on the basis of a meaningful combination of Confucianism and Taoism. In philosophy, there are two schools of metaphysics, namely, gui-wu and chong-you, centered on whether there are questions or not.
