# Supplementary material for: Psycho-Linguistic Changes Associated With Historical Celebrities in Henan Using Classical Chinese Big Data
Source: Front Psychol. 2021 Sep 14;12:648677. doi: 10.3389/fpsyg.2021.648677 (PMC8476835; doi:10.3389/fpsyg.2021.648677)
Supplement: Supplementary file 2 [file Table_1.DOCX]

Supplementary Material

# Supplementary Data

**A list of 47 historical celebrities from Henan：**

| **序号** | **历史时期** | **姓名** | **介绍** | **性别** | **出生年月** |
| --- | --- | --- | --- | --- | --- |
| 1 | 1 | Lao Tzu | the founder of the Taoist school. born in Kuxian county of Chu (today's Luyi, Henan) during the Spring and Autumn Period | Male | Unknown |
| 2 | 1 | Zi Gong | the ancestor of Chinese Confucian merchants, born in Wei (present-day Jun County, Henan) | Male | BC520- Unknown |
| 3 | 1 | Mozi | Mozi, an outstanding thinker, educator and founder of mohist school, born in Lushan, Henan province | Male | BC468-BC376 |
| 4 | 1 | Li Fan | a famous statesman, strategist and merchant in ancient China, was honored as the saint of Commerce by later generations. born in Wan of Chu State (now Nanyang, Henan Province). | Male | Unknown |
| 5 | 1 | Qin Su | a famous strategist, born in Xuanli, Luoyang (today's Luoyang, Henan) during the Eastern Zhou Dynasty. | Male | Unknown -BC284 |
| 6 | 1 | Yi Zhang | a political strategist in the Warring States Period, born in the State of Wei (now Kaifeng, Henan province). | Male | Unknown -BC310 |
| 7 | 1 | Zi Chan | A well-known politician, thinker, and pioneer of Legalism, Zichan, born in Zheng Guo (now Xinzheng, Henan). | Male | Unknown -BC520 |
| 8 | 1 | Zhuangzi | Famous thinker, philosopher, Taoist school representative Zhuangzi, Meng of Song state, born in Min Quan County, Henan province. Also called ZhuangZhou. | Male | BC369-BC286 |
| 9 | 1 | Buwei Lu | A famous businessman and statesman,born in Puyang city of Wei (in present-day Yanshi county, Henan Province). His tomb is located in Yanshi City, Luoyang | Male | Unknown -BC235 |
| 10 | 1 | Han Fei | the master of legalist thought, was a man of Korea in the late Warring States Period (now xinzheng, Henan province, has a different opinion) | Male | BC280-BC233 |
| 11 | 1 | Yang Shang | an outstanding statesman and reformer in ancient China, = born in the middle of the Warring States Period in Wei (now Neihuang, Henan) | Male | BC390-BC338 |
| 12 | 2 | Ping Chen | the founder of the Western Han Dynasty and a famous strategist, was a man of Yangwu (now Yuan Yang, Henan, has a different opinion) | Male | Unknown -BC178 |
| 13 | 2 | Liang Zhang | one of the three outstanding statesmen and strategists in the late Qin and early Han Dynasties, was a native of Baofeng, Henan province. has a different opinion. | Male | Unknown -BC185 |
| 14 | 2 | Cuo Zhao | The Western Han Dynasty statesman, a native of Yingchuan (now Yuzhou, Henan), has the tomb on the west side of the Martyrs Cemetery in Xuchang. | Male | BC200-BC154 |
| 15 | 2 | Shizhi Zhang | who was famous for his impartial law enforcement, was born in Duyang (now south of Henan) in the Western Han Dynasty. There are Shizhi Zhang’s Tomb and Shizhi Zhang’s Temple in Fangcheng nowy. | Male | Unknown |
| 16 | 2 | Yi Jia | an outstanding political commentator and writer in the early Western Han Dynasty, was from Luoyang. | Male | BC200-BC168 |
| 17 | 2 | Yi Feng | the founding hero of the Eastern Han Dynasty and a famous military strategist, born in Yingchuan Father City (now Baofeng, Henan), and his tomb is in Baofeng County today. | Male | Unknown -34 |
| 18 | 2 | Shao Yuan | Shu Yuan and Shao Yuan, the heroes of the late Han Dynasty, were both from Runan Ruyang (now Shangshui, Henan). | Male | Unknown -202 |
| 19 | 2 | You Xun | Father Xun Yi (yí) is Xun Yu’s uncle brother | Male | 157-214 |
| 20 | 2 | Yu Xun | An outstanding politician, strategist, Cao Cao adviser Xun or, from Yingchuan Yingyin (now Xuchang, Henan). | Male | 162-212 |
| 21 | 2 | Yong Cai | Famous writer and calligrapher, Liuyu people of Chen(now Qixian County, Henan Province, or Weishi County). | Male | 132-192 |
| 22 | 2 | Heng Zhang | An outstanding scientist and writer in ancient times, known as the saint of science, born in Xi'e, Nanyang (now Shiqiao Town, Nanyang City, Henan) | Male | 78-139 |
| 23 | 3 | Yi Sima | An outstanding statesman and military strategist in the Wei Kingdom, he founder of the Western Jin Dynasty, born in Wen County, Hanoi (now Wen County, Henan) | Male | 179-251 |
| 24 | 3 | Ai Deng | an outstanding military strategist and general of the Wei Kingdom, born in Jiyang (now Xinye, Henan) in Yiyang. | Male | 197-264 |
| 25 | 3 | Ji Ruan | a writer of the Wei Dynasty and one of the seven sages of the bamboo forest, born in the Wei clan of Chenliu (now Weishi, Henan). There are Ji Ruan tomb and Ji Ruan shouting platform in current Weishi | Male | 210-263 |
| 26 | 3 | Yao Zhong | a famous calligrapher and politician in Cao Wei .He was the ancestor of calligraphy history and the originator of regular script,born in Yingchuan Changshe (now Changge, Henan). | Male | 151-230 |
| 27 | 3 | Jun Wang | born in Hu County, Hongnong County (now Lingbao City, Henan Province). Famous general in the Western Jin Dynasty. | Male | 206-285 |
| 28 | 3 | Xuan Xie | a famous general in the Eastern Jin Dynasty, born in Taikang, Henan. | Male | 343-388 |
| 29 | 3 | Yue Pan | a writer and beautiful man, born in Zhongmu (now Zhongmu County, Henan). | Male | 247-300 |
| 30 | 3 | Lingyun Xie | the founder of the School of Landscape Poetry in the history of Chinese literature, and the great historical traveler,born in Taikang. | Male | 385-433 |
| 31 | 3 | Ye Fan | The famous historian and writer, born in Shunyang (now Xichuan, Henan) in the Southern Song Dynasty. | Male | 398-445 |
| 32 | 3 | Zhen Fan | a thinker and outstanding atheist in the Southern Dynasties between Qi and Liang, was from Nanxiang Wuyin (now Biyang, Henan) (with different opinions) | Male | 450-510 |
| 33 | 3 | Yan Jiang | Famous minister of Qiliang in the Southern Dynasties, famous writer, with allusions such as the brilliant writing of flowers, Jiang Lang, etc., a native of Jiyang Kaocheng (now Henan Minquan) | Male | 444-505 |
| 34 | 4 | Chong Yao | a famous minister known as the prime minister at the time of rescue, was from Xiashi, Shanzhou (now Shanxian County, Henan). | Male | 650-721 |
| 35 | 4 | Rengui Liu | Minister and famous military general, from Zhou Weishi (now Weishi, Henan). | Male | 601-685 |
| 36 | 4 | Jing Wu | born in Bianzhou Junyi (now Kaifeng, Henan). Minister of the Tang Dynasty, a famous historian. | Male | 670-749 |
| 37 | 4 | Xun Zhang | Born in Nanyang, Dengzhou in "New Tang Book". Famous minister in the middle of Tang Dynasty. | Male | 709-757 |
| 38 | 4 | Shuo Zhang | Literary and politician,originally from Fanyang and later moved to Luoyang, Henan. | Male | 667-730 |
| 39 | 4 | Juyi Bai | The well-known realist poet,born in Xinzheng, Henan. | Male | 772-846 |
| 40 | 4 | Jie Yuan | pioneer of the ancient prose movement and writer Yuan Jie, born in Lushan, Henan. | Male | 719-772 |
| 41 | 4 | Yu Han | Outstanding writer, thinker and politician, from Heyang (now Mengzhou, Henan), Hanoi (with different opinions) | Male | 768-824 |
| 42 | 4 | Yuxi Liu | The famous poet, known as a poet, born in Luoyang (with different terms). | Male | 772-842 |
| 43 | 4 | Jian Wang | written as Zhongchu, born in Yingchuan, Xuzhou (now Xuchang City, Henan Province), minister and poet of the Tang Dynasty. | Male | 767-830 |
| 44 | 4 | Zhen Yuan | Famous poet Yuan Zhen, from Luoyang | Male | 779-831 |
| 45 | 4 | Pu Zhao | The famous minister, born in Ji County, Youzhou (now Beijing), and later moved to Changshan (now Zhengding, Hebei) and Luoyang (now Luoyang, Henan). | Male | 922-992 |
| 46 | 4 | Guang Sima | a politician, writer, and historian, born in Sushui Township, Xia County, Shanxi Province, and born in Guangshan County, Henan Province. | Male | 1019-1086 |
| 47 | 4 | Fei Yue | a famous strategist, military strategist, and anti-gold warrior in my country’s history, born in Tangyin (now Tangyin, Henan Province) in Xiangzhou in the Northern Song | Male | 1103-1142 |

Note:1=Spring-Autumn Period and Warring States Period;2= Han Dynasty;3= The Three Kingdoms and Jin and Southern and Northern Dynasties;4= Tang Dynasty;5= Song Dynasty
